# Identification and Functional Verification of the Glycosyltransferase Gene Family Involved in Flavonoid Synthesis in *Rubus chingii* Hu

**DOI:** 10.3390/plants13101390

**Published:** 2024-05-16

**Authors:** Yujie Shi, Zhen Chen, Mingkai Shen, Qianfan Li, Shunli Wang, Jingyong Jiang, Wei Zeng

**Affiliations:** 1 Zhejiang Provincial Key Laboratory of Plant Evolutionary Ecology and Conservation, College of Life Sciences, Taizhou University, Taizhou 318000, China; shiyujie@tzc.edu.cn (Y.S.); chenzh@tzc.edu.cn (Z.C.); 17285849240@163.com (S.W.); 2 State Key Laboratory of Subtropical Silviculture, College of Forestry and Biotechnology, Zhejiang A&F University, Hangzhou 311300, China; 2367851070@stu.zafu.edu.cn (M.S.); qianfanli@stu.zafu.edu.cn (Q.L.); 3 Institute of Horticulture, Taizhou Academy of Agricultural Sciences, Linhai 317000, China; jjy5971@163.com

**Keywords:** *Rubus chingii*, UGTs, phylogeny, flavonoid, functional analysis

## Abstract

Glycosylation is catalyzed by UDP-glycosyltransferase (*UGT*) and plays an important role in enriching the diversity of flavonoids. *Rubus* plants contain a lot of natural flavonoid glycosides, which are important plants with a homology of medicine and food. However, information about the *Rubus UGT* gene family is very limited. In this study, we carried out genome-wide analysis and identified the 172, 121, 130, 121 *UGT* genes in *R. chingii*, *R. corchorifolius*, *R. idaeus*, and *R. occidentalis*, respectively, and divided them into 18 groups. The analysis of the protein motif and gene structure showed that there were structural and functional conservations in the same group, but there were differences among different groups. Gene replication analysis showed that raspberry and dicotyledons had a higher homology. The expansion of the *UGTs* gene family was mainly driven by tandem replication events, and experienced purified selection during the long evolution of the raspberry. Cis-acting element analysis showed that they were related to plant growth and development, hormone regulation, and stress response. In addition, according to a comprehensive analysis of the co-expression network constructed by transcriptome data and phylogenetic homology, *RchUGT169* was identified as a flavonoid glucosyltransferase. Through the transient expression in tobacco, it was verified that *RchUGT169* could catalyze the conversion of kaempferol and quercetin to the corresponding flavonoid glycosides. In conclusion, this research enriched the understanding of the diversity of UGTs in *Rubus* and determined that RcUGT169 can catalyze flavonoids.

## 1. Introduction

*Rubus* L. is one of the genera with the most species in Rosaceae, which contains a large number of economically important species, such as black raspberry (*Rubus occidentalis* L.), blackberry (*Rubus fruticosus* L.), red raspberry (*Rubus idaeus* L.), Chinese raspberry (*Rubus chingii* Hu.), and Shanmei (*Rubus corchorifolius* L.) [1,2]. *R. chingii* is endemic to China, mainly distributed in East China (Zhejiang, Fujian, Jiangsu, and Anhui provinces), so it is also known as “Huadong Fu-Pen-Zi” [3]. The fruit can be eaten raw or made into jam and wine and can also be planted as an ornamental plant. In addition, *R. chingii* can also be used in medicine, and it is the only species of Chinese raspberry listed in the Chinese Pharmacopoeia [3]. Its medicinal part is its immature dry fruit, which contains a large number of flavonoids, terpenoids, polysaccharides, and other substances, especially flavonoids [4,5], which can play a strong role in anti-oxidation, anti-inflammation, anti-cancer, anti-hypertension and the protection of blood vessels [3,6,7]. Therefore, raspberry is a kind of medicine and a food homologous variety, which has great prospects in research and development. 

Flavonoids are secondary metabolites that are widely found in fruits and medicinal plants, mainly in the form of flavonoid glycosides, and their biosynthesis depends on the catalysis of glycosyltransferases (GTs, EC 2.4.x.y) [8,9]. GTs exist in all organisms and are responsible for transferring the glycosyl portion from the activated donor to the receptor, thus participating in various biological metabolic processes [10]. So far, 116 GT subfamilies have been identified in Carbohydrate-Active enZYmes (CAZY) database [11]. Glycosyltransferase 1 (GT1) is the largest family, with UDP-glucose as the main sugar donor, followed by UDP-galactose, UDP-rhamnose, UDP-xylose, and UDP-glucuronic acid, which are mainly responsible for the glycosylation of plant secondary metabolites [12,13]. Therefore, the GT1 family is also known as UDP glycosyltransferases (UGTs). In terms of structure, the plant *UGT* gene is highly conserved, which usually contains a structure of 44 conserved amino acid sequences at the C-terminal, which is called the Plant Secondary Product Glycosyltransferase (PSPG box) conserved domain; the N-terminal sequence of PSPG is related to the recognition and binding of sugar receptors, showing a relative diversity, indicating that the catalytic domain may be involved in the binding of a variety of sugar receptors [14,15].

The *UGTs* of plants not only participate in the synthesis of plant secondary metabolites, but also have a variety of biological functions, such as a role in plant antitoxin and defense mechanisms, the response to abiotic stress, and in the regulation of plant hormones [13,16,17]. *UGT* genes have been identified in many plants, including *Arabidopsis thaliana* (121) [9], *Populus trichocarpa* (191) [18], wheat (179) [19], *Gossypium raimondii* (142) [20], *Quercus robur* (244) [21], apple (299) [22], *Morella rubra* (152) [23], alfalfa (90) [24], and so on. Up to now, there have been few studies on the *UGT* family of *R. chingii*, only mentioned in the publication of the Chinese raspberry genome [2], and the number of identified *UGT* genes may be incomplete. Therefore, in order to better study the function of *UGTs* in *R. chingii,* we will systematically identify and verify the UGT genes at a genome-wide level.

The publication of the genome at the raspberry chromosome level makes it possible to explore the characteristics of the *UGT* genes family in this horticultural crop that also has the concomitant function of both medicine and foodstuff. In this study, we identified *UGTs* based on the genomes of *R. chingii* and related species (*R. corchorifolius*, *R. idaeus*, and *R. occidentalis*), and further analyzed the *UGTs* from *R. chingii*, including their sequence characteristics, phylogenetic relationship, gene structure, chromosome distribution, gene collinearity, and cis-acting elements. Finally, through phylogenetic analysis of the raspberry *UGT* gene and the known functional *UGT* gene in different plants, combined with the expression characteristics of flavonoid structural genes in different developmental stages of raspberry fruit, a co-expression network was constructed to comprehensively identify flavonoid glycosyltransferase-related genes. The function of genes was verified by enzyme activity experiments, which provided a basis for the further study of the molecular mechanism of *UGTs* in the secondary metabolite biosynthesis pathway of raspberry.

## 2. Material and Methods

### 2.1. Identification of the UGT Gene Family in Rubus

The identification and analysis of the *UGT* gene family were based on the whole genome of *Rubus chingii*, *R. corchorifolius*, *R. idaeus*, and *R. occidentalis*. The genome data of *R. chingii*, *R. idaeus*, and *R. occidentalis* were downloaded from the GENOME DATABASE FOR ROSACEAE (GDR, https://www.rosaceae.org/, accessed on 20 October 2023), the *R. corchorifolius* genome data were obtained from China National Center Bioinformation (CNCB, https://www.cncb.ac.cn/, accessed on 20 October 2023), and other genome data (*Arabidopsis thaliana*, *Oryza sativa*, *Zea mays*, *Solanum lycopersicum* and *Populus trichocarpa*) were downloaded from the Phytozme 13 database (https://phytozome-next.jgi.doe.gov/, accessed on 20 October 2023) [25]. Firstly, all the protein sequences of the *UGT* gene family in *A. thaliana* were obtained from the CAZY Database (http://www.cazy.org/, accessed on 20 October 2023). Then, these sequences were used as query sequences to search all possible *UGT* sequences in four plants by the BLASTP 2.15.0 program (E-value ≤ 1 × 10^−5^). At the same time, the hidden markov model (HMM) of the UDP-glucoronosyl and UDP-glucosyl transferase domain (UDPGT, PF00201) was obtained from Pfam database (https://www.ebi.ac.uk/interpro/entry/pfam/, accessed on 20 October 2023) to perform the HMMER program [26] with the same E-value. Finally, all possible UGT protein sequences of the four species were submitted to CDD (https://www.ncbi.nlm.nih.gov/Structure/bwrpsb/bwrpsb.cgi, accessed on 20 October 2023), SMART (http://smart.embl.de/, accessed on 20 October 2023) and InterPro (https://www.ebi.ac.uk/interpro/, accessed on 20 October 2023) databases for conservative domain verification, and the candidate UGTs with an incomplete PSPG box were deleted to obtain accurate *UGT* gene family members.

The physical and chemical properties of each member of the *UGT* gene family were predicted by ExPASy online software (https://web.expasy.org/protparam/, accessed on 20 October 2023) [27], including the Amino Acid (aa), Molecular Weight (Mw), Isoelectric Point (PI), Instability Index, Aliphatic Index, and the Grand Average of Hydropathicity (GRAVY). The subcellular localization of UGT family members were predicted by WoLF PSORT (https://www.genscript.com/wolf-psort.html, accessed on 20 October 2023).

### 2.2. Phylogenetic Analysis and the Classification of the UGT Gene Family in Rubus

Multiple alignment of the UGT protein sequences of *Rubus* and *A. thaliana* was carried out by MUSCLE 3.8.31 tool, and a phylogenetic tree was constructed based on the neighbor-joining (NJ) method using MEGA 11 software [28] with 1000 bootstraps. Moreover, the *UGT* gene family of *Rubus* were classified according to the UGT subfamily in *A. thaliana* [9]. Each protein sequence was named according to its position on the chromosome and was visualized using the ChiPlot online program (https://www.chiplot.online/ accessed on 20 October 2023) [29].

### 2.3. Structure and Conserved Motif Analysis of the UGT Gene Family in Rubus

According to the gene annotation file of *Rubus*, the structural maps of the *UGT* genes were constructed using TBtools No. 2.007 [30]. In addition, the conserved motifs of the UGT protein were identified using the MEME online software (https://meme-suite.org/meme/tools/meme, accessed on 25 October 2023). The width of the conserved sites was set to 6–50, and the maximum number of conserved sequences was set to 10. Finally, TBtools was used to visualize the Exon-Intron structure, conserved motif, conserved domain, and the evolutionary tree of four *Rubus UGT* gene families, respectively.

### 2.4. Chromosome Mapping and Gene Duplication Analysis of UGT Genes

The location information of the corresponding *UGT* genes were obtained from the genome annotation file of *Rubus* and visualized by TBtools. The repetitive events and collinearity of genes in four *Rubus* plants and among different plants were analyzed by MCScanX software, and the maps were drawn by Circos v0.69-9 software. Moreover, the values of Ka, Ks, and ratio between replicating gene pairs were calculated using the KaKs_Calculator package [31]. The approximate occurrence time (Mya) of duplication events was estimated by using T = Ks/2λ × 10^−6^ (λ = 1.5 × 10^−8^ substitutions/synonymous site) [32].

### 2.5. Cis-Acting Regulatory Element Analysis

In order to reveal the possible regulatory elements of *UGTs* promoter in *R. chingii*, we extracted the 2000-bp DAN sequence of upstream of the start codons of the *UGT* genes and uploaded them to the PlantCARE database (http://bioinformatics.psb.ugent.be/webtools/plantcare/html/, accessed on 27 October 2023) for analysis. The Cis-Acting regulatory elements were classified according to different functions, and the results were visualized via heat map and bar chart.

### 2.6. Transcriptomic and RT-qPCR Analysis of UGTs Gene in R. chingii 

Using the transcriptomic data from fruits of four developmental stages (Big Green (BG, 21 DAY), Green-to-Yellow (GY, 42 DAY), Yellow-to-Orange (YO, 48 DAY), Red (Re, 54 DAY) published by our research group [33], FPKM values were used to estimate the expression level of *RchUGTs*. The FPKM values of all periods were normalized by Log2, and the expression heat map was drawn using TBtools.

In order to verify the expression pattern of the selected genes, the total RNA was extracted from the fruits of raspberry at four developmental stages using OminiPlant RNA Kit (CW2598, CWBIO, Taizhou, China). Then, the total RNA extracted were converted into cDNA using one-step reverse transcription kit (G592, ABM, Hangzhou, China). The *RchActin* gene was used as an internal reference gene and cDNA as a template for RT-qPCR amplification. The primers of RT-qPCR were designed by Primer5 software and synthesized by Tsingke Biotech Co., Ltd. (Beijing, China), and all primers sequences accessed in Table S2. PCR was carried out via CFX96 instrument (Bio-Rad, Hercules, CA, USA), and the results were analyzed using 2^−△△Ct^ algorithm. Each replicate was performed more than five times.

### 2.7. Protein Interaction Network Analysis of the RchUGTs

Because the *UGT* genes of *R. chingii* were not available in String database (https://cn.string-db.org/, accessed on 28 October 2023), in order to study the interaction between the UGT proteins, we searched the homologous genes of *RchUGT*s in *A. thaliana* by OrthoVenn2 online program (https://orthovenn2.bioinfotoolkits.net/home, accessed on 28 October 2023). The protein–protein interaction network was visualized using Cytoscape software v3.10.2 [34].

### 2.8. Functional Prediction of the RchUGTs Involved in Flavonoid Biosynthesis

To identify the *RchUGTs* involved in flavonoid biosynthesis, we screened the *RchUGT* genes using two methods. Firstly, according to the research method of Zhao et al. [8], the 58 sequences of other plant UGT proteins with known functions were obtained (Table S6). Using these protein sequences as a reference, the UGT genes related to flavonoid biosynthesis pathway in *R. chingii* were screened by constructing the ML phylogenetic tree by IQ-TREE [35] as dataset 1.

In addition, we used the fruit transcriptome data of four different developmental stages to obtain the FPKM values of structural genes (*CHS*, *PAL, 4CL, F3H, DFR, ANS, ANR, LAR,* and *FLS*) related to flavonoid biosynthesis. The Chiplot online program was used to analyze the correlation between these genes and *RchUGTs,* and the *RchUGT* genes with significant correlation were screened as dataset 2. Finally, dataset 1 and dataset 2 were intersected to obtain the final candidate genes.

### 2.9. Protein Recombination and Subcellular Localization

The coding sequences of *RchUGT169* and *VENUS* (with YFP marker) were recombined into the KpnⅠ and BamHⅠ sites in the pFUERTE vector [36], pFUERTE-*RchUGT169-VENUS* constructs were transformed into *Agrobacterium tumefaciens* (AGL1 + pSoup). *RchUGT169* yellow fluorescent protein (YFP) was used for the transient transformation of tobacco leaves, and the pFUERTE-*VENUS* vector was used as the control. A laser scanning confocal microscope (IX83-FV3000, OLYMPUS, Tokyo, Japan) was used to observe the instantaneous transformation of tobacco leaves. OLYMPUS FV31S-SW v2.6 software was used to record images. The argon ion laser lines used were 488 nm for YFP and chlorophyll, and the fluorescence of YFP and chlorophyll was detected at 495–530 nm and 650–680 nm, respectively.

### 2.10. Protein Extraction and Purification

All the tobacco leaves transformed by *RchUGT169* were collected and pre-cooled 1 × Hepes buffer solution was added for full grinding. The ground homogenate was filtered into a 50 mL centrifuge tube with Miracloth (Millipore, Billerica, MA, USA) and centrifuged at a low speed (at 4 °C, 4000 rpm for 10 min). Then, the supernatant was transferred to another centrifuge tube for ultra-high-speed centrifugation (Optima XPN-100, BECKMAN COULTER, Brea, CA, USA), and the parameters were 100,000× *g*, 4 °C, 35 min. Finally, the supernatant was removed, then 400 μL 1 × Hepes buffer was added, and the homogenate was obtained as total plant protein.

The total plant protein was divided into two parts, one was used for the Western Blotting (WB) experiment, and the VENUS was used as control. The other was purified by GFP-Trap magnetic agarose (gtma, Chromotek, Planegg, Germany), and the purified protein was used to detect the enzyme activity.

### 2.11. Enzyme Activity Detection of the RchUGT169 Recombinant Protein

Using UDP-Glc as a glycosyl donor and quercetin or kaempferol as the receptors, the reaction system (100 μL) to verify RchUGT169 protein activity was as follows: experimental groups (4 μL UDP-Glc, kaempferol or quercetin 1 μL, RchUGT169 protein was dissolved with 100 μL 1 × Hepes buffer) and the control group (4 μL UDP-Glc, kaempferol or quercetin 1 μL, RchUGT169 protein (inactivated at 90 °C) was dissolved with 100 μL 1 × Hepes buffer). The above solutions were mixed and reacted at 25 °C, 1300 rpm for 4 h in a metal bath, respectively, and the supernatants were filtered with 0.22 um filter membrane for LC-MS/MS (Thermo Scientific Q Exactive, Waltham, MA, USA) detection at 350 nm. The chromatographic column was Agilent ZORBAX SB-C18 column (250 × 4.6 mm, 5 μm) (Agilent, Santa Clara, CA, USA), the column temperature was 30 °C, the injection volume was 2 μL, the mobile phase A was 0.1% formic acid aqueous solution (*v*/*v*), and the mobile phase B was 100% acetonitrile. The gradient elution procedure was as follows: 0–2 min, 30% B; 2–20 min, 30% B; 20–25 min, 100% B; 25–30 min, 30% B. The experimental conditions of mass spectrometry were as follows: the ion source was ESI, the negative ionization mode was used, and the acquisition mode was full scan, auto MS/MS.

### 2.12. Statistical Analyses and Solution Preparation

The IBM SPSS 26 software was used to analyze the data and compare the differences among groups. Duncan multiple comparison in one-way analysis of variance (ANOVA) was used to detect the significant difference, and the significance level was *p* < 0.05. 

Quercetin (Q817162, MACKLIN, Shanghai, China) and kaempferol (K812226, MACKLIN, China) were mixed with methanol to form the mother liquor of 100 mM, respectively, and UDP-Glc (U850073, MACKLIN, China) was prepared with ddH_2_O as the mother liquor of 50 mM. 2 × Hepes (1 L) formula: first dissolve Hepes powder (BS106, BIOSHARP, Shanghai, China) in ddH_2_O, adjust the PH to 6.8, add 273.6 g sucrose, then add 20 mL 0.5 M MgCl_2_ and MnCl_2_, fixed volume to 1 L. The above mother liquids were stored at −20 °C. In addition, when using the Hepes buffer (PH 6.8), one piece of protease inhibitor (BL630B, BIOSHARP, China) should be added to each 50 mL 1 × Hepes solution.

## 3. Results

### 3.1. Identification of the UGT Gene Family in Rubus

Through HMMER search and BLASTP program, we screened the genes with UDPGT conserved domain as the candidate genes of the *Rubus UGT* gene family. Then, using multiple databases for verification and the deletion of incomplete sequences, a total of 172, 121, 130, and 121 *UGT* genes were identified in the whole genomes of *R. chingii* (*RchUGTs*), *R. corchorifolius* (*RcoUGTs*), *R. idaeus* (*RidUGTs*), and *R. occidentalis* (*RocUGTs*) for further analysis (Table S1). The protein length encoded by these *UGT* genes ranges from 124 (*RchUGT80*) to 2397 (*RocUGT79*) aa. The molecular weight ranges from 13.62 (*RchUGT80*) to 266.59 (*RocUGT79*) kDa, and the theoretical isoelectric point varied from 4.59 (*RocUGT36*) to 9.54 (*RchUGT*46). In *R. chingii*, *R. corchorifolius, R. idaeus,* and *R. occidentalis*, there were 42 (24.4%), 33 (27.3%), 30 (23.1%), and 42 (34.7%) proteins with an instability index less than 40, respectively, indicating that most of the UGT proteins in the four *Rubus* were unstable. The aliphatic index ranges from 72.46 (*RocUGT51*) to 109.49 (*RocUGT51*). The GRAVY of 16 (*RchUGT*s), 13 (*RcoUGT*s), 19 (*RidUGT*s) and 21 (*RocUGTs*) proteins were greater than 0, respectively, indicating that most of the UGT proteins were hydrophilic. Subcellular localization prediction showed that 96 and 59 proteins were located in the chloroplast and cytoplasm in *R. chingii,* 58 and 36 proteins in the chloroplast and cytoplasm in *R. corchorifolius*, 67 and 40 proteins in the chloroplast and cytoplasm in *R. idaeus*, 58 and 41 proteins in the chloroplast and cytoplasm in *R. idaeus*, and a small number of other proteins in nucleus, extracellular, cell membrane, plasma membrane, and so on. Studying the physical and chemical properties of UGT proteins are helpful to understanding its unique biological function.

### 3.2. Phylogenetic Analysis of UGT Family Genes

**Figure 1:**
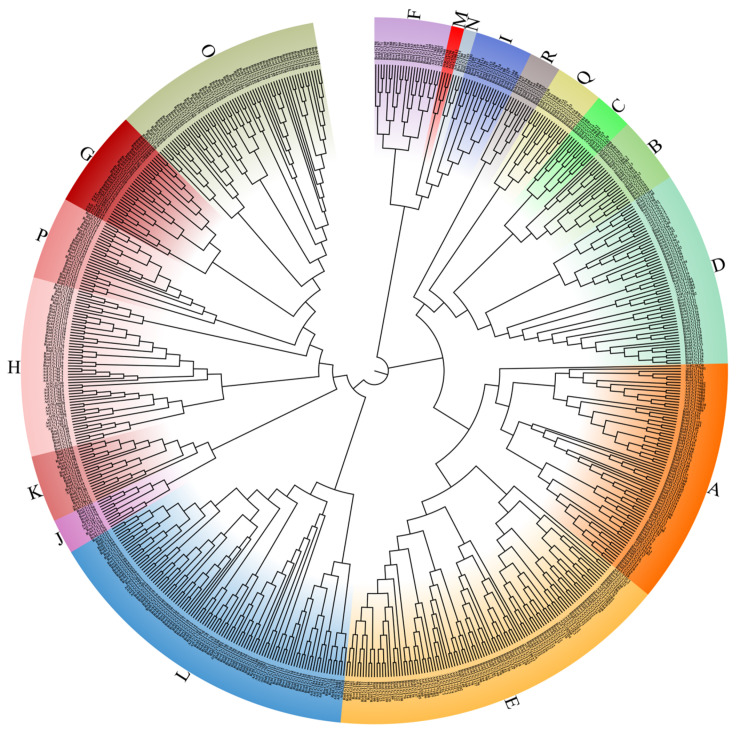
Phylogenetic tree of the *UGT* gene family. The genealogical tree of UGT proteins from *A. thaliana*, *R. chingii* (*RchUGTs*), *R. corchorifolius* (*RcoUGTs*), *R. idaeus* (*RidUGTs*), and *R. occidentalis* (*RocUGTs*). Various colors and capital letters indicate the different groups of *UGT* genes.

In order to further study the evolutionary relationship of *UGT* genes in *Rubus*, a phylogenetic tree was constructed by using *UGT* genes from four *Rubus* and *A. thaliana* plants. Raspberry *UGT* genes were classified according to the 14 groups of *A. thaliana* (A-N) *UGT* genes (Figure 1). There were 16, 16, 17, and 17 groups identified in *R. chingii*, *R. corchorifolius*, *R. idaeus*, and *R. occidentalis*, respectively, and group M only existed in *A. thaliana*, while four new groups (O, P, Q, and R) were also found in *Rubus*. The *UGT* genes of four *Rubus* species were unevenly distributed among groups, and there were great differences among species. The groups L and O had the most members in *R. chingii* (31 and 32), and group L also occupied most members in *R. idaeus* (26) and *R. occidentalis* (17), respectively, and group E in *R. corchorifolius* (24) had the most members. Furthermore, compared with the other three *Rubus* plants UGTs, *the RchUGTs and RchUGTs lacked* group G and N, respectively. There were the most *UGT* genes in *R. chingii* (172), which were much more than *R. corchorifolius* (121), *R. idaeus* (130), and *R. occidentalis* (121). These results suggest that *R. chingii* may have experienced more complex evolutionary patterns than the other three species of *Rubus* plants.

### 3.3. UGTs Gene Structure, Conserved Motif, and Domain

**Figure 2:**
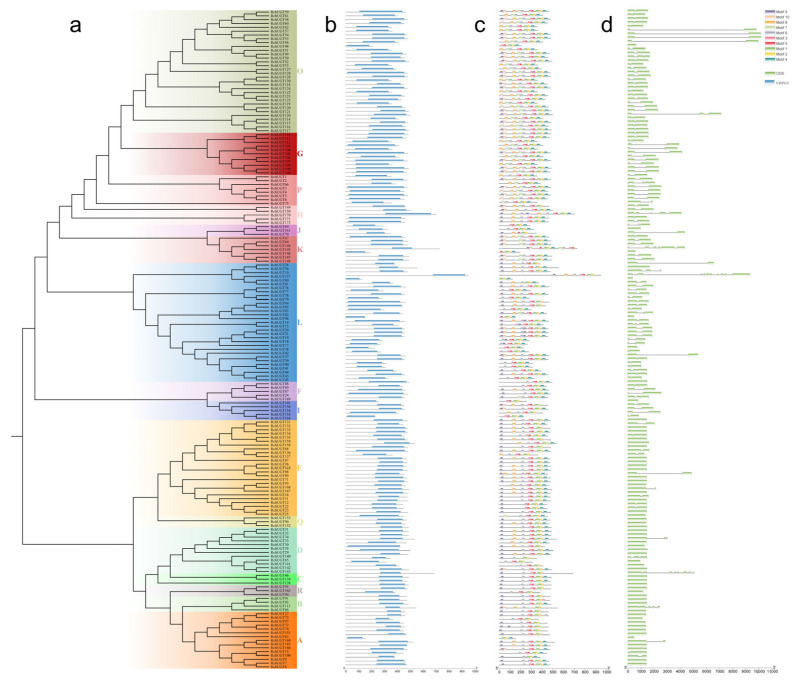
Phylogenetic tree, conserved protein structure, conserved motif and gene structure analysis of the *RchUGTs*. (**a**) The evolution tree was created by proteins sequences of 172 *RchUGTs*, various colors and capital letters indicate different groups of the *RchUGT* gene. (**b**) Conserved protein structure analysis of *RchUGTs*. (**c**) Conserved motif position of *RchUGTs*. (**d**) Exon-intron structure analysis of *RchUGTs*.

Phylogenetic trees were constructed based on *RchUGT*, *RcoUGT*, *RidUGT*, and *RocUGT* proteins. The phylogenetic relationships among different members of *UGTs* in four plants of *Rubus* were further analyzed, and their conserved domains, protein motifs, and gene exon-intron structures were predicted (Figure 2 and Figures S1–S3). There was a complete UDPGT domain in all UGT proteins (Figure 2b and Figures S1–S3). At the same time, a total of 10 MEME prediction motifs were identified and annotated with InterPro database. Motifs 1 and 2 in *RchUGTs*, motifs 1 and 3 in *RcoUGTs*, *RidUGTs*, and *RocUGTs* all relate to a 44 amino acid conserved sequence in the C-terminal (Plant Secondary Product Glycosyltransferase, PSPG), which were found in all plant UGT proteins (Figure S4). The results showed that the identification of members of the UGT gene family in four kinds of plants was reliable.

In UGT proteins, the number of motifs ranges from 3 to 10, and most UGT members contained all 10 motifs (Figure 2c and Figures S1–S3). In *RchUGTs*, motifs 9 and 4 were located at the N-terminal and C-terminal of the UGTs sequence, respectively. In *RcoUGTs*, motifs 4 and 6 were located at the N-terminal and C-terminal of the UGT sequence, respectively. In *RidUGTs*, motifs 5 and 10 were located at the N-terminal and C-terminal of the UGT sequence, respectively. In *RocUGTs*, motifs 4 and 7 were located at the N-terminal and C-terminal of the UGT sequence, respectively. The sequences of these motifs were similar for different species. Moreover, motif 7 was missing in the A/B/C/D/E/Q/R groups of *RchUGTs*. The overall results showed that the type and position of conserved motifs among UGT members were consistent with the phylogenetic relationship among groups (Figure 2a).

The characteristics of gene structure were an important basis for analyzing the phylogeny of gene families. Exon-intron analysis showed that the intron number of *RchUGT* genes varied from 0 to 10 (Figure 2d), of which only one member (*RchUGT157*) had the most introns, 53 members lacked introns, and most of the members lacking introns were located in the bottom-most branch (A/B/C/D/E/Q/R). In addition, similar results were found in three other raspberries (Figures S1–S3). These results indicate that the *RchUGTs* genes were quite different among different groups, but it was quite conservative in the bottom branch, and there was a similar evolution pattern among different *Rubus* plants.

### 3.4. Chromosome Distribution and Gene Duplication Analysis of UGT Family Genes in Rubus

According to the genomic location of 544 *UGT* genes on their respective chromosomes of four raspberries, the chromosomal distribution of *RchUGTs*, *RcoUGTs*, *RidUGTs* and *RocUGTs* were determined (Figure S5). Among the four raspberries, the *UGT* genes were located on all 7 chromosomes, indicating that the chromosome distribution of the *UGT* gene family in raspberry were unbiased. It was worth noting that the distribution of *UGT* genes on different chromosomes were uneven. The *UGT* genes of the four raspberries had the most distribution on Chr6, with the largest number of genes being 43, 31, 25, and 29, respectively, while the number of *UGT* genes on Chr1 of *R. chingii*, Chr5 of *R. corchorifolius*, Chr1 of *R. idaeus*, and Chr4 of *R. occidentalis* were the least, and there were only 15, 11, 15, and 11 genes in each chromosome. Moreover, *RchUGTs* were quite different from the other three raspberries on Chr 2 and 3. There were two big gene clusters on Chr 2 and 3, respectively, which contain a large number of *UGT* genes. The results showed that there were similarities and differences in the evolution of the *UGT* gene among the four raspberries, mainly in the *RchUGTs*, so the following analysis was dominated by the *UGTs* gene of *R. chingii*.

**Figure 3:**
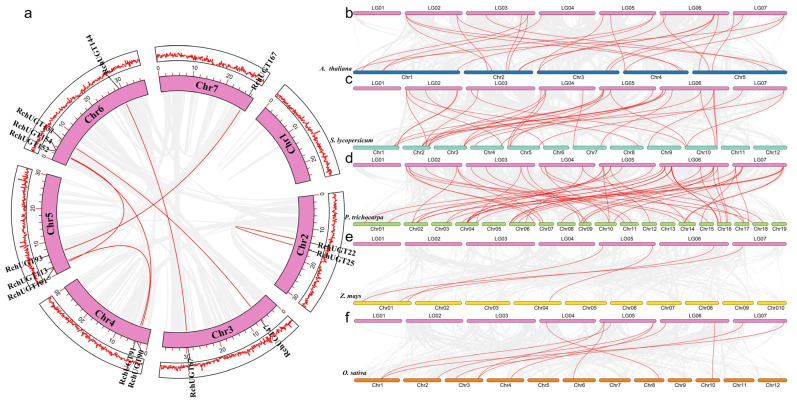
Collinear analysis of the *RchUGT* gene family. (**a**) The collinearity of raspberry genes within species. The circle indicates that the seven chromosomes of raspberry have different markers. The gray and red wired genes show all collinear blocks and fragment doubling events. The outermost layer of the circle represents the gene density corresponding to each chromosome. (**b**–**f**) Genetic collinearity between *R. chingii* and different species, including *A. thaliana*, *S. lycopersicum*, *P. trichocarpa*, *Z. mays*, and *O. sativa*. Rectangles of different colors represent chromosomes from different species. The grey and red linker genes show the collinear relationships between all collinear blocks and *UGTs*, respectively.

In order to explore the amplification mechanism of the *RchUGT* genes in the genome of *R. chingii*, genomic collinearity and evolution were studied. We identified 7 segmental repeats and 112 tandem repeats in the *RchUGTs* gene (Figure 3a and Table S3). Tandem repeats were more than segmental repeats, indicating that tandem repeats played a crucial role in the amplification of *RchUGTs* in evolution. Furthermore, the analysis showed that the highest number of duplications occurred on Chr6, which may be the main reason for the higher number of *RchUGT* genes on Chr6. The amplification mechanism of *RcoUGT*, *RidUGT*, and *RocUGT* genes were similar to that of the *RchUGT* genes (Figure S6). The ratio of Ka to Ks represents the direction in which genes were selected during the evolutionary process. Through calculation, it was found that the all Ka/Ks values < 1, indicating that the *UGT* gene family had undergone purification selection in the long evolutionary engineering process. Furthermore, the differentiation time of these genes were estimated, and the differentiation time of the *UGT* genes ranged from 2.96 (*RocUGT86*-*RocUGT98*) to 138.49 (*RocUGT66*-*RocUGT96*) MYA (Table S4). However, the average differentiation time difference among the four species was small, with 58.49 (*RchUGTs*), 53.39 (*RcoUGTs*), 51.92 (*RidUGTs*), and 56.55 (*RocUGTs*), respectively.

It is well known that members of the gene family evolved from a common ancestor. Therefore, we drew collinear map of the raspberry *UGT* gene and five other plants. The collinear map showed that there were 24, 10, 58, 4, and 33 pairs of homologous genes between *R. chingii* and *A. thaliana*, *O. sativa*, *P. trichocarpa*, *Z. mays,* and *S. lycopersicum*, respectively (Figure 3b–f). Among them, there were 6, 1, 18, and 10 *RchUGTs* genes in raspberry, and at least two pairs of homologous genes in *A. thaliana*, *O. sativa*, *P. trichocarpa,* and *S. lycopersicum*, respectively, suggesting that these genes might play an important role in the phylogeny of the *UGT* gene family. In addition, the homologous gene pairs between raspberry and dicotyledonous plants (*A. thaliana, P. trichocarpa*, and *S. lycopersicum*) were greatly more than those between raspberry and monocotyledon plants (*O. sativa* and *Z. mays*), and there were the most homologous genes with a woody plant poplar, which indicated that the *UGTs* gene might be involved in the differentiation of dicotyledonous plants and had higher homology in higher woody plants.

### 3.5. Cis-Acting Elements Analysis of UGT Genes in R. chignii

**Figure 4:**
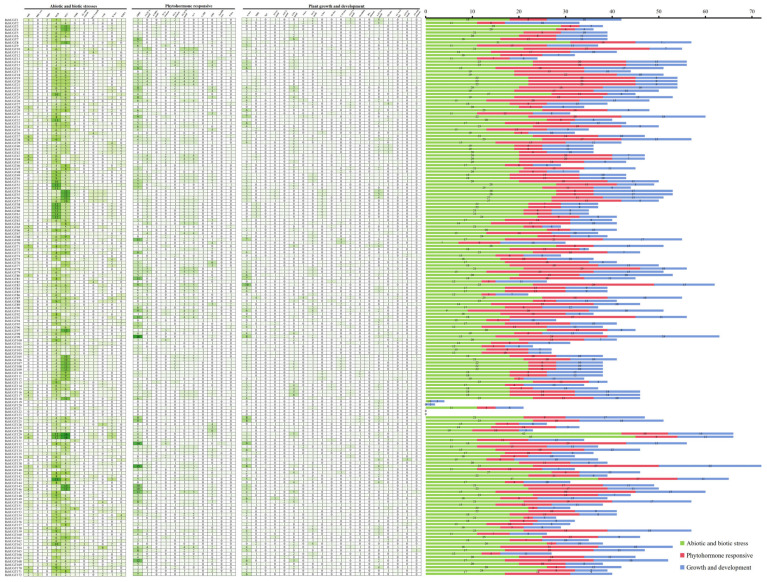
The analysis of cis-acting elements of the gene promoter of *RchUGTs*. The gradient color in the heat map represents the number of cis-acting elements of *RchUGT*s. The color histogram represents the total number of cis-acting elements in each category.

The upstream promoter region (~2000 bp) was obtained from the raspberry genome sequence to understand the transcriptional regulation characteristics of the *RchUGTs*. The cis-acting elements of the *RchUGTs* promoter were explored by using PLANTCARE database, and 41 types of cis-acting elements were observed (Figure 4). These elements were involved in abiotic and biotic stresses, phytohormone responsiveness, and plant growth and development. 

Among the cis-acting elements of phytohormone responsiveness, abscisic acid-, MeJA-, gibberellin-, salicylic acid-, and auxin- responsive elements existed in the promoter of *RchUGTs*, respectively, among which abscisic acid- (617) and MeJA- (530) responsive elements occupied most response elements. There were many elements related to stress, including ARE, LTR, MBS, and TC-rich, which were involved in anaerobic induction, low-temperature responsiveness, drought-inducibility, and defense stress responses, respectively. This suggested that the gene expression of these promoter elements might be regulated by environmental stress, abscisic acid and MeJA. In plant development elements, there were a large number of elements involved in light responsiveness, cis-acting regulatory element related to meristem expression, involved in circadian control, cis-acting regulatory element involved in seed-specific regulation, cis-regulatory element involved in endosperm expression and so on, indicating that *RchUGTs* played an important role in regulating plant growth and development. In addition, the number of cis-acting elements in the *RchUGTs* was uneven, ranging from 0 (*RchUGT122/123*) to 72 (*RchUGT139*). Among them, *RchUGT122* and *RchUGT123* may be nonfunctional pseudogenes, while *RchUGT139* may be involved in a variety of biological network regulation pathways.

### 3.6. Transcriptome and RT-qPCR Analysis of RchUGTs during Fruit Development

**Figure 5:**
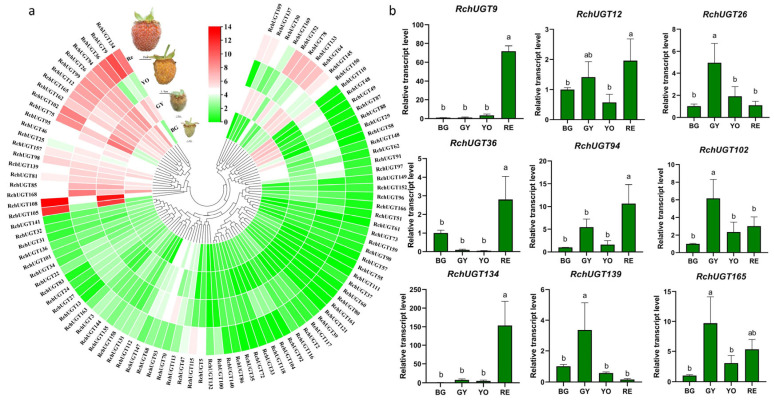
The expression patterns of *RchUGTs* in four stages of fruits development and corresponding RT-qPCR analysis. (**a**) RNA-seq results of *RchUGTs* in four stages of fruits development, and photos of fruits in four stages. (**b**) RT-qPCR results of *RchUGTs* in four stages of fruits development. BG (big green, 21 DPA), GY (green-to-yellow, 42 DPA), YO (yellow-to-orange, 48 DAP), and Re (red, 54 DPA). Different lowercase letters represent significant differences (*p* < 0.05) between different groups.

After filtering the low expression RNA-seq data, a total of 100 *RchUGT*s were used to further describe the expression profiles of four fruit development stages (Figure 5a). We constructed a cluster heat map to explore the expression characteristics of raspberry at four fruit development stages via normalizing the expression. The results showed that the expression of common 43 *RchUGT* genes fluctuated obviously in different developmental stages, mainly from the E group (9), and the rest belonged to A (3), B (1), C (2), D (2), F (3), G (5), H (1), K (3), L (5), O (5), P (1), and R (3) groups. These results suggested that these 43 *RchUGT* genes might play a more important role in glycosylation during raspberry fruit development.

In order to verify the accuracy of transcriptome data, 22 genes were randomly selected from 43 genes, and the transcriptional levels of the above 22 *RchUGTs* were analyzed using the RT-qPCR method (Figure 5b and Figure S7). The results showed that the expression patterns of most genes were consistent with those of RNA-seq analysis, which supported the reliability of transcriptome data analysis. Among them, the expression of *RchUGT9/36/94/134* genes were the highest in Re, which was significantly higher than that in the other three periods; the expression of *RchUGT26/102/139/46/75/81/85/95/98/99/162* in GY were significantly higher than that in other periods; while *RchUGT12/165* were highly expressed in GY and Re; and *RchUGT25/157168* were highly expressed in BG and GY. These RT-qPCR expression patterns showed that in the GY stage of fruit development, *RchUGTs* were the most active, followed by the Re stage.

### 3.7. Protein Interaction Network Analysis of the RchUGTs

**Figure 6:**
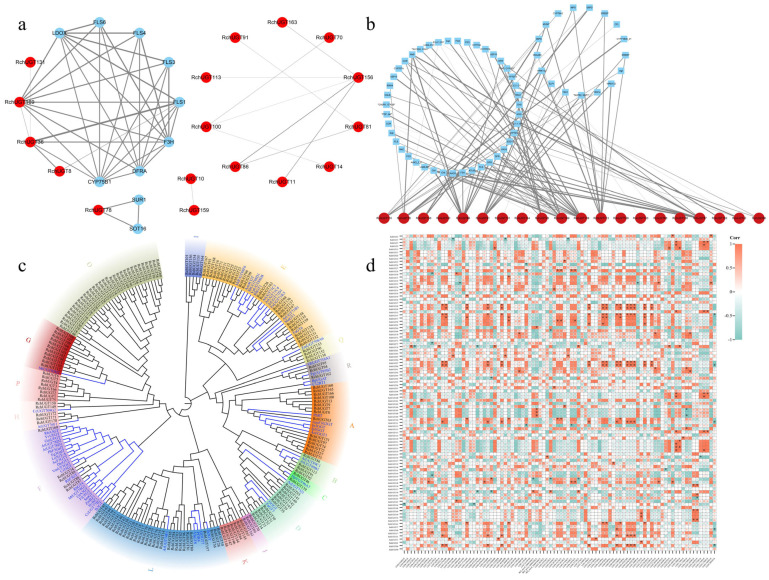
Protein interaction network, phylogeny, and correlation analysis of *RchUGTs* related to the flavonoid. (**a**) Construction of the *RchUGTs* protein interaction network based on *Arabidopsis thaliana* homologous genes. (**b**) Network interaction analysis of 20 flavonoid *RchUGTs* genes and related genes. (**c**) The phylogenetic tree based on 172 *RchUGTs* and 58 *UGTs* with flavonoid receptors using the ML method, various colors and capital letters indicate different groups of the *RchUGT* gene. (**d**) Correlation analysis heat map between the *RchUGTs* transcription level and flavonoid biosynthesis-related genes. * represents *p* < 0.05; ** represents *p* < 0.01; *** represents *p* < 0.001.

To further understand the function and role of the *RchUGTs* protein, a protein interaction network was constructed through the STRING database with *Arabidopsis* homologue genes as reference (Table S5). The results of the protein interaction network showed that *RchUGT169* was related to a large number of flavonoid structure genes (*FLS1/3/4/6*, *F3H*, *CYP75B1, LDOX*, and *DFRA*), and *RchUGT36/8* were also related to flavonoid structure genes (Figure 6a). Furthermore, interactions among a large number of members of *RchUGTs* had been observed, indicating that there was functional collaboration among them. These results suggest that the *RchUGTs* protein may be involved in the biosynthesis pathway of flavonoids by interacting with target proteins.

### 3.8. Prediction of Candidate RchUGTs Related to Flavonoid Biosynthesis

In order to further explore the *UGTs* related to flavonoid glycosylation in raspberry, we first constructed a phylogenetic tree based on 172 *RchUGTs* and 58 *UGTs* with flavonoid receptors using the ML method (Figure 6c and Table S6). The results showed that 73 *RchUGTs* had a close phylogenetic relationship with known flavonoid UGT proteins in plants and might have similar glycosylation functions in flavonoid *UGTs*. These *RchUGTs,* which were specific to flavonoids, were mainly distributed in the A/B/C/D/E/F/G/H/L/Q/R groups, and the E group had the most *RchUGTs* (15). Moreover, *RchUGTs* were also divided into OG1, OG8 (GGT), OG7, OG14 (5GT), and OG23 (3GT), according to the catalytic position and function of flavonoids (Table S7). In addition, we also analyzed the correlation between *RchUGTs* transcription level and flavonoid biosynthesis related genes (*CHS*, *PAL*, *4CL*, *F3H*, *DFR*, *ANS*, *ANR*, *LAR,* and *FLS*). The results showed that 74 members of *RchUGTs* (43%) were significantly correlated with genes related to flavonoid metabolism, and the correlation coefficients were all greater than 0.9 (Figure 6d). 

Combining the common screening of the above two methods, 20 *RchUGTs* were found to be closely related to flavonoid biosynthesis, and the network map was established between 20 *RchUGTs* and related genes according to the results of KEGG annotation (Figure 6b). The flavonoid *UGTs* genes were further screened by comparing the 20 genes with the results of the interaction of the above proteins. Finally, *RchUGT169* (*LG07.63*) was obtained, which belongs to the OG23 (3GT) group, and had a high homology with flavonoid UGTs, and had great differences in expression at the different stages of fruit development (Figure S8), which may be involved in the glycosylation of flavonoids during raspberry fruit development.

### 3.9. Detection of the Subcellular Localization and Enzyme Activity of RchUGT169 Recombinant Protein

**Figure 7:**
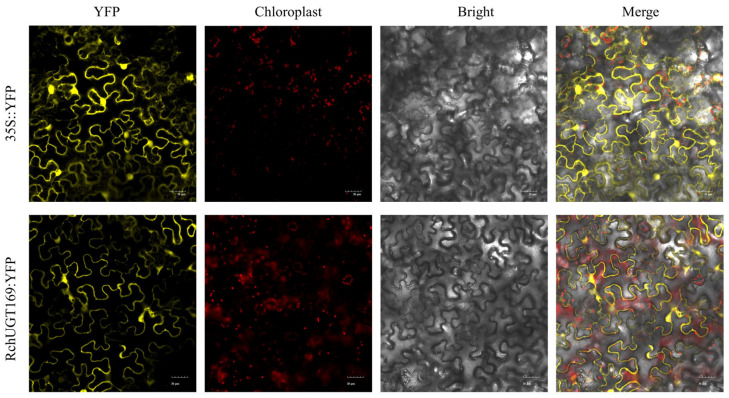
Subcellular localization of the empty vector (35S::YFP, CK) and RchUGT169-YFP fusion protein.

Using *Rubus* cDNA as template, the *RchUGT169* gene was cloned. The ORF of the *RchUGT169* gene was 1398 nucleotides and encodes a protein of 466 amino acids (Figure S9). Then, the *RchUGT169*-YFP vector was constructed and transiently expressed in tobacco, and its subcellular localization was determined. The *RchUGT169*-YFP fusion protein may be located in the cytoplasm (Figure 7), while the 35S::YFP control was distributed in the whole cell.

**Figure 8:**
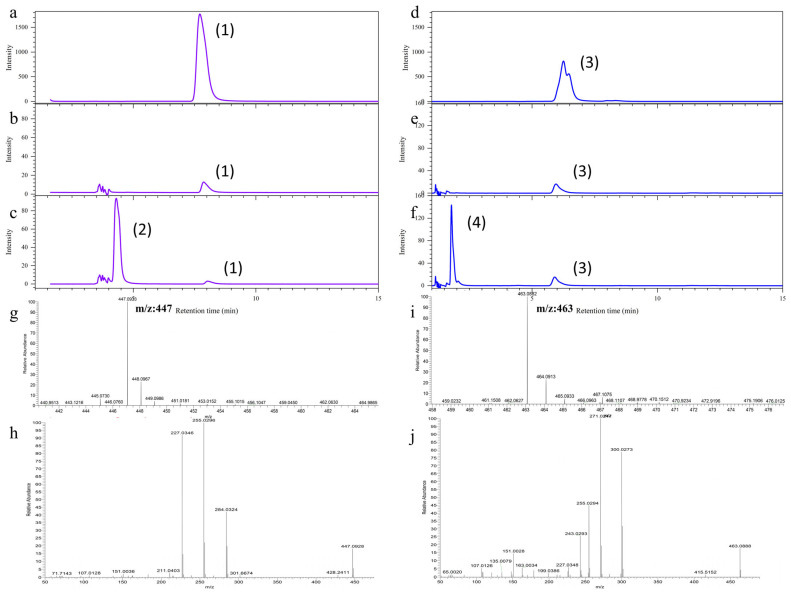
Enzyme activity analysis of the RchUGT169 recombinant protein. (**a**,**d**) are the detection of kaempferol and quercetin, respectively; (**b**,**e**) are the control groups of enzymatic reaction of kaempferol and quercetin; (**c**,**f**) are the experimental groups of enzymatic reaction of kaempferol and quercetin; (**g**,**i**) are the MS1 of products (2) and (4); (**h**,**j**) are MS2 of products (2) and (4). Products (1), (2), (3), and (4) are kaempferol, kaempferol glucoside, quercetin, and quercetin glucoside, respectively.

The WB experiment was performed to verify the expression of the RchUGT169 recombinant protein, and the results showed that the molecular weight of the RchUGT169 recombinant protein was ~77 kDa (Figure S9). Furthermore, the function of RchUGT169 was verified using the enzyme activity method. The results showed that RchUGT169 could transfer UPD-Glc to kaempferol or quercetin (Figure 8a–f). According to the fragment information, the products of *m*/*z* 447 and *m*/*z* 463 were identified as kaempferol glucoside (Figure 8g,h) and quercetin glucoside (Figure 8i,j).

## 4. Discussion

Glycosyltransferases (GTs) are encoded by a large polygene family and widely exist in plants. It is responsible for the glycosylation of plant secondary metabolites and determines the diversity of metabolites. As the largest gene family in GTs, UGTs have been identified in model plants (*A. thaliana* [9], rices [37], poplars [18], etc.) and a variety of horticultural plants (pomegranates [8], grapes [38], bayberries [23], peaches [39], apples [22], kiwifruits [40] and strawberries [41], etc.). However, raspberry, an important plant homologous to medicine and food, has not been studied. In order to deepen our understanding of the UGT family in raspberry, we analyzed the whole genome of raspberry and three related species of *Rubus*. In addition, based on the characteristics of gene expression during phylogeny and fruit ripening, the UGTs involved in flavonoid biosynthesis were identified and classified, and verified by enzyme activity experiments, which laid a foundation for the follow-up study of the catalytic function of raspberry UGTs.

### 4.1. Characterization of UGT Genes in Rubus

In this study, 172, 121, 130, and 121 *UGT* genes were identified from 4 *Rubus* plants, accounting for 0.54%, 0.45%, 0.23%, and 0.36% of the total genes, respectively. The proportion of *UGT* genes in *R. chingii* was higher than that in *A. thaliana* (0.44%) [9], pomegranate (0.4%) [8] and soybean (0.26%) [42], but lower than that in peach (0.6%) [39]. Through phylogenetic analysis, all *UGT* genes of *Rubus* were divided into 18 groups (Figure 1), which were 13 highly conserved groups (A-L, N) and 4 newly discovered groups (O, P, Q, R). According to previous research, *UGT* genes were first divided into A-N groups, mainly based on Cruciferae plants [12,42], such as *Arabidopsis*, *Brassica napus*, *B. oleraca*, *B. rapa*; then O and P groups were found in most plants, such as peach [39], *Citrus grandis* [43], *Gossypium arboretum* [20], *Glycine max* [44], *Nelumbo nucifera* [45], etc. Finally, Q and R groups were found in a few plants, such as *Camellia sinensis* [46], *G. barbadense* [47], Z. mays [48], *Punica granatum* [8] and so on, but they all contained only one group. It was worth noting that Q and R groups simultaneous existed in apples of Rosaceae [49], which was consistent with the results of raspberry in this study. The result indicated that Q and R groups might make an important contribution to the glycosylation of metabolites in Rosaceae.

Generally speaking, A, D, E, G, and L were considered to be the fastest evolving groups in the evolution of higher plants [44]. Among the four *Rubus* plants, the number of *RchUGTs* were far more than that of the other three *Rubus* plants, mainly concentrated in A (9%), D (7%), E (14%), G (6%), L (18%), and O (19%) groups, indicating that the members of these five groups expanded faster than those of the other groups in the process of plant evolution, and had similar results to those of pomegranate [8], tea [46], and *Dendrobium catenatum* [50]; contrary to the results of peach [39], the number of *UGTs* in Rosaceae was not all caused by the obvious expansion of G and M groups, because M group was missing in all the *Rubus* plants of Rosaceae, but 4 new groups (O, P, Q, R) were formed, which might replace the lost function of M group, and there were more *UGTs* genes in O group.

The position, phase, loss, and acquisition events of introns are important clues to understand evolution [51]. The intron map of 172 *UGT* genes in raspberry showed that 31% of the members lacked introns, which was lower than that of most plants, such as pomegranate (36%) [8], peach (43%) [39], *A. thaliana* (58%) [9], and maize (60%) [48]. Ten introns were identified in *RchUGTs*, and motifs 1 and 2 encoding the UGT domain were found in all identified *UGT* genes. Moreover, although the closely related *UGT* members seem to have similar protein motifs and gene structures, some *UGT* genes are different from other members of the group. For example, the UGT members lacking introns were distributed in the A/B/C/D/E/Q/R groups, and these groups were clustered into a large branch, resulting in greater differentiation with the rest of the groups (Figure 2). Therefore, these specific differences may lead to the differentiation of raspberry UGT function.

Among the 172 *RchUGT* genes identified; all were located on 7 chromosomes. These genes usually exist in clusters on chromosomes and show a high sequence similarity in the same cluster, which was consistent with the situation of *UGT* genes in *D. catenatum* and cotton [20,50]. Gene replication is a common phenomenon in the process of plant evolution, which helps to produce genes with new functions. The formation of polygene families mainly comes from whole-genome duplication (WGD) events and gene replication in specific regions of chromosomes [52]. In this study, we found 7 segmental duplications and 112 tandem duplications, respectively, that was, tandem duplication was the main driving force of *RchUGTs* expansion (Figure 3), which was also consistent with previous findings in *A. thaliana* [53], grapes [54], *Epimedium pubescens* [55], and pomegranate [8]. However, some contrary results had been reported, such as no tandem replication was observed in soybean, and a series of segmental replication led to *UGT* expansion [42]. The segmental replication was also the main gene replication event in pears [56]. Taken together, these results suggest that the expansion of the *UGT* family, driven by replication events, is species-specific.

### 4.2. Identification and Verification of the RchUGTs Involved in Flavonoid Biosynthesis

The spatio-temporal expression pattern of genes can provide powerful supplementary information for genome analysis and help to screen new candidate genes for the glycosylation of secondary metabolites. In raspberry, 100 *UGT* genes were highly expressed during fruit development (Figure 5). Through phylogenetic analysis with known functional UGT, combined with the co-expression network analysis of flavonoid related structural genes, the candidate UGT involved in flavonoid glycoside synthesis was screened. The results suggest that *RchUGT169* may be involved in the formation of flavonoid 3-O-glycosides (Figure 6).

In previous studies, most of the members of the *UGT* family in group F were identified to have flavonols and anthocyanidins 3-O-glycosyltransferase activity [57,58,59]. For example, *VvGT5* and *VvGT6* of group F from grapes were identified as flavonol 3-O-glucuronosyltransferase and bifunctional flavonol 3-O-glucosyltransferase/galactosyltransferase [60]. In waxberry, four *UGT* members (*MrUGT78R1/78R2/78W1* and *MrUFGT*) in group F were identified as flavonoid 3-O-glycosyltransferases involved in the accumulation of different flavonoid glycosides [61]. In tea, *CsUGT78A14* and *CsUGT78A15* from group F were reported to be responsible for the biosynthesis of flavonol 3-O-glucoside and flavonol 3-O-galactoside, respectively [46]. Furthermore, *PpUGT78A2* in group F of peach was also identified as flavonoid 3-O-glycosyltransferase involved in different glycosylation of flavonols and anthocyanidins [62]. In raspberry, through phylogenetic analysis, *RchUGT169* gene belonged to group F and was classified as OG23 (3GT subfamily), which was considered to be a flavonoid 3-O-glycosyltransferase [14]. This gene was highly expressed during the color conversion period of fruit ripening (GY and Re), indicating that it may be the main catalytic enzyme of flavonoid 3-O-glycosylation during raspberry fruit development. Moreover, for the sugar receptors of UGT, the substrates of UGT are diverse, and the same UGT may catalyze many types of substrate glycosylation. For example, in this study, *RchUGT169* can catalyze two common flavonol compounds (kaempferol and quercetin), so it was further identified as flavonol 3-O-glucosyltransferase (Figure 8).

## 5. Conclusions

In this study, the whole genome of the *UGT* gene family in *Rubus* was analyzed, including the gene structure, conserved motif, chromosome distribution, gene replication mode, cis-acting element, and expression pattern. Through polygenic analysis, 172, 121, 130, and 121 *UGTs* genes were identified in *R. chingii*, *R. corchorifolius*, *R. idaeus*, and *R. occidentalis*, respectively, and were into 18 groups. All *UGT* genes were located on 7 chromosomes, and most *UGT* genes of 4 *Rubus* plants were distributed on Chr 6. Gene replication analysis showed that they were mainly driven by tandem replication events. Expression profile analysis showed that *RchUGTs* played an important role in fruit development and ripening, and the *RchUGT169* gene was closely related to the biological process of flavonoids. Finally, the enzyme activity experiment confirmed that *RchUGT169* could catalyze the glycosylation of kaempferol and quercetin. The results will provide a new idea for *Rubus* fruit ripening and screening suitable genes related to flavonoid biosynthesis and contribute to the molecular biology study of *Rubus* plants.

## Data Availability

The RNA-seq data in this study were deposited in the NCBI Sequence Read Archive under the BioProject with the accession number PRJNA671545.

## Supplementary Materials

The following supporting information can be downloaded at: https://www.mdpi.com/article/10.3390/plants13101390/s1, Figures S1–S3: Phylogenetic tree, conserved protein structure, conserved motif and gene structure analysis of *RcoUGTs*; Figure S4: SeqLogo of the Motif 1 to 10 among four plants of *Rubus*; Figure S5: Chromosome Mapping of *UGTs* Gene in four *Rubus* species; Figure S6: Collinear analysis of *UGTs* gene family from *R. corchorifolius* (a), *R. idaeus* (b) and *R. occidentalis* (c); Figure S7: RT-qPCR results of *RchUGTs* in four stages of fruits development. BG (big green, 21 DPA), GY (green-to-yellow, 42 DPA), YO (yellow-to-orange, 48 DAP) and Re (red, 54 DPA); Figure S8: The expression patterns of *RchUGT169* in four stages of fruits development; Figure S9: Full-length CDS cloning of *RchUGT169* gene of raspberry and SDS-PAGE of RchUGT169 recombinant protein; Table S1: Physical and chemical properties of proteins encoded by UGT of four *Rubus*; Table S2: Primer sequences used RT-PCR; Table S3: The replication ways of UGTs gene in *Rubus*; Table S4: Evolutionary selection and differentiation time of *UGTs* Gene in *Rubus*; Table S5: Homologous genes and functional description of raspberry and *Arabidopsis thaliana*; Table S6: The 58 known *UGTs* genes with flavonoids as substrate receptors; Table S7: Screening of *RchUGTs* related to flavonoid UGT Glycosylation based on phylogenetic Tree.
