# Supplementary material for: Identification and Functional Verification of the Glycosyltransferase Gene Family Involved in Flavonoid Synthesis in Rubus chingii Hu
Source: Plants (Basel). 2024 May 16;13(10):1390. doi: 10.3390/plants13101390 (PMC11125054; doi:10.3390/plants13101390)
Supplement: Supplementary file 1 [file plants-13-01390-s001.zip › Supplementary files (Fig S1-9).pdf]

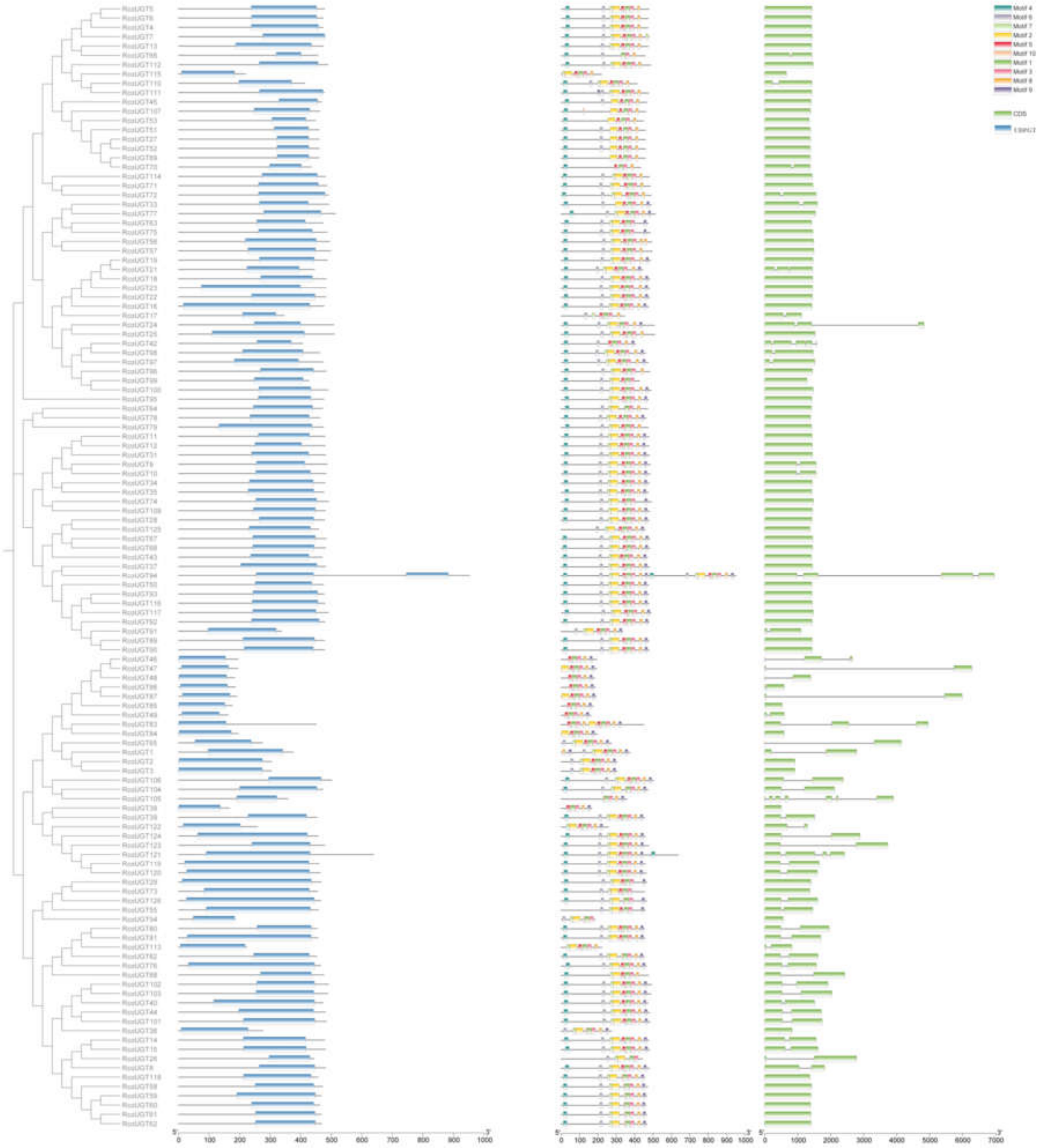

Figure S1 Phylogenetic tree, conserved protein structure, conserved motif and gene structure analysis of *RcoUGTs*.

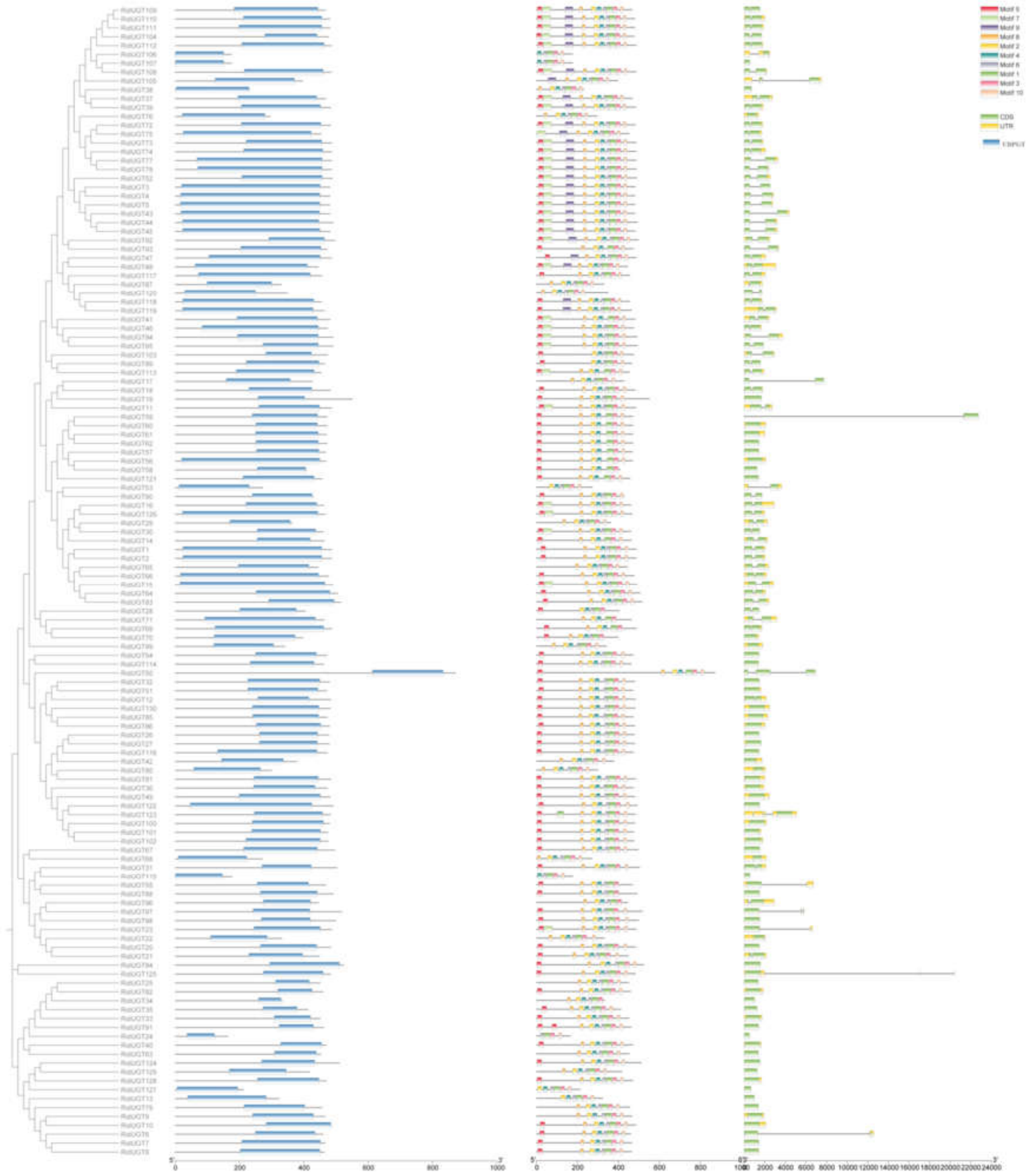

Figure S2 Phylogenetic tree, conserved protein structure, conserved motif and gene structure analysis of *RidUGTs*.

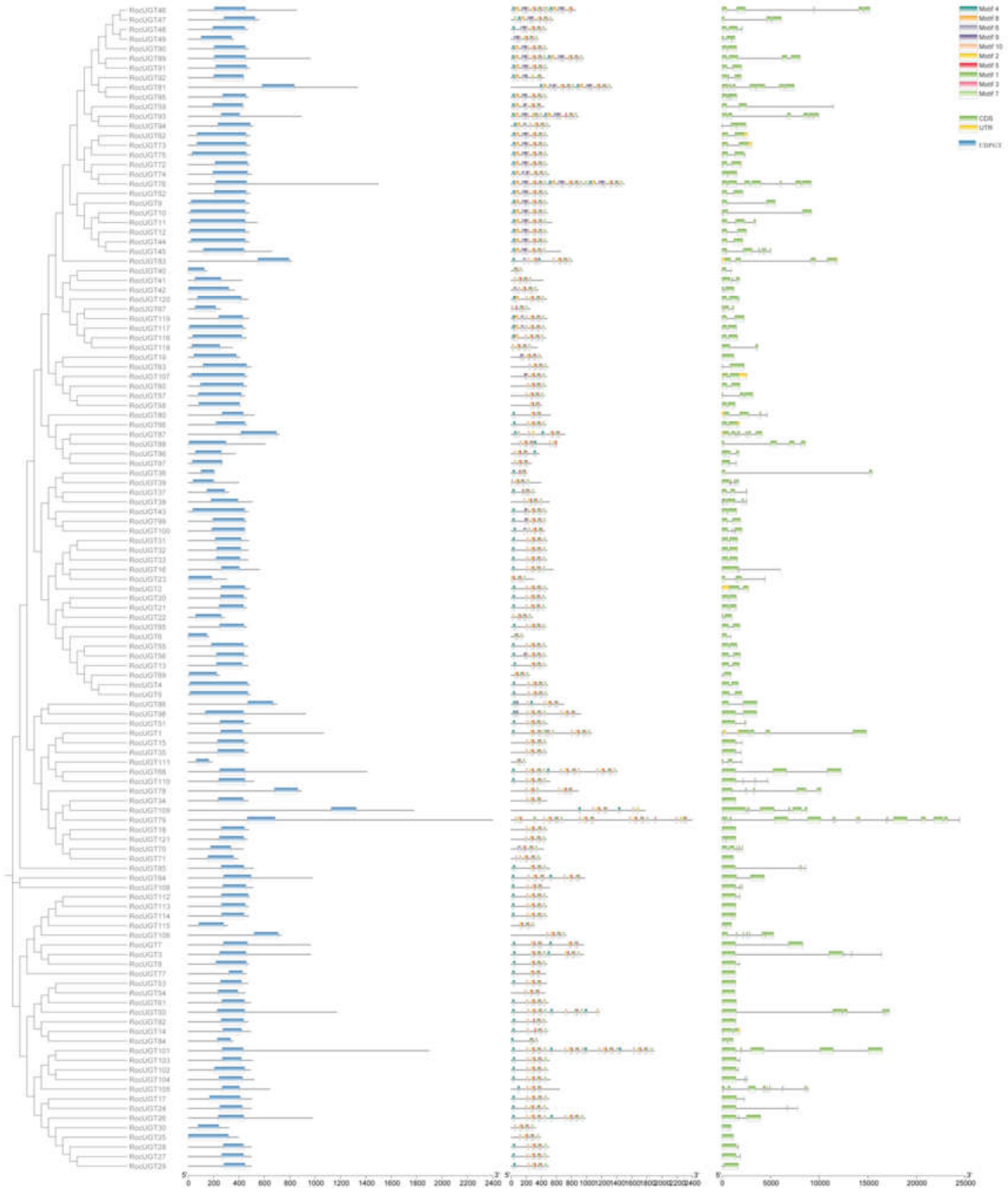

Figure S3 Phylogenetic tree, conserved protein structure, conserved motif and gene structure analysis of *RocUGTs*.

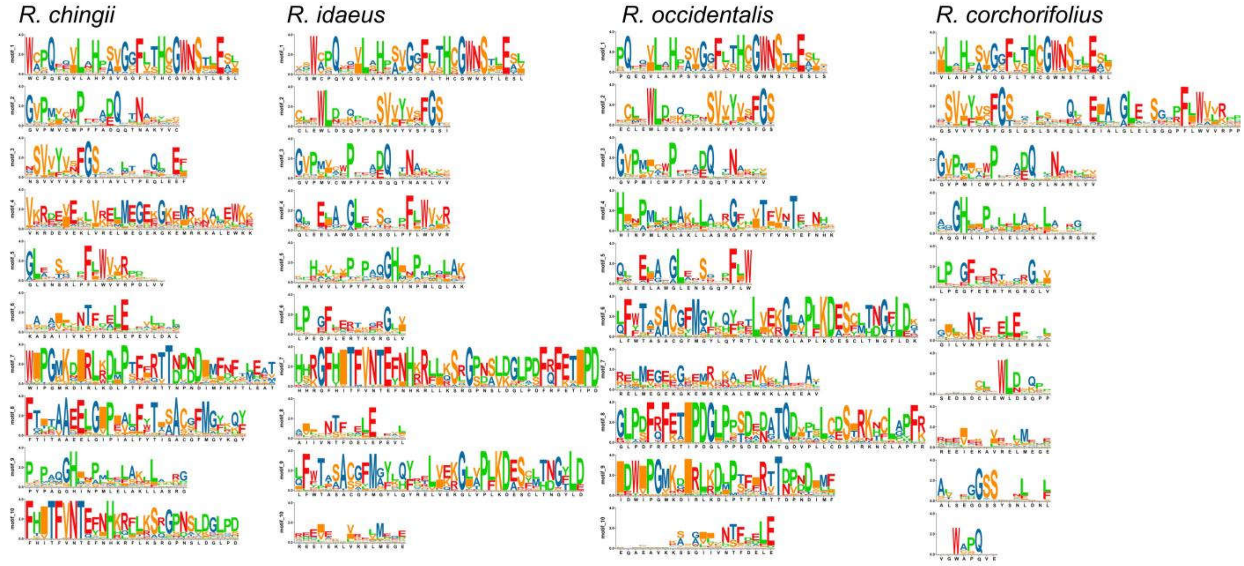

Figure S4 SeqLogo of the Motif 1 to 10 among four plants of *Rubus.*

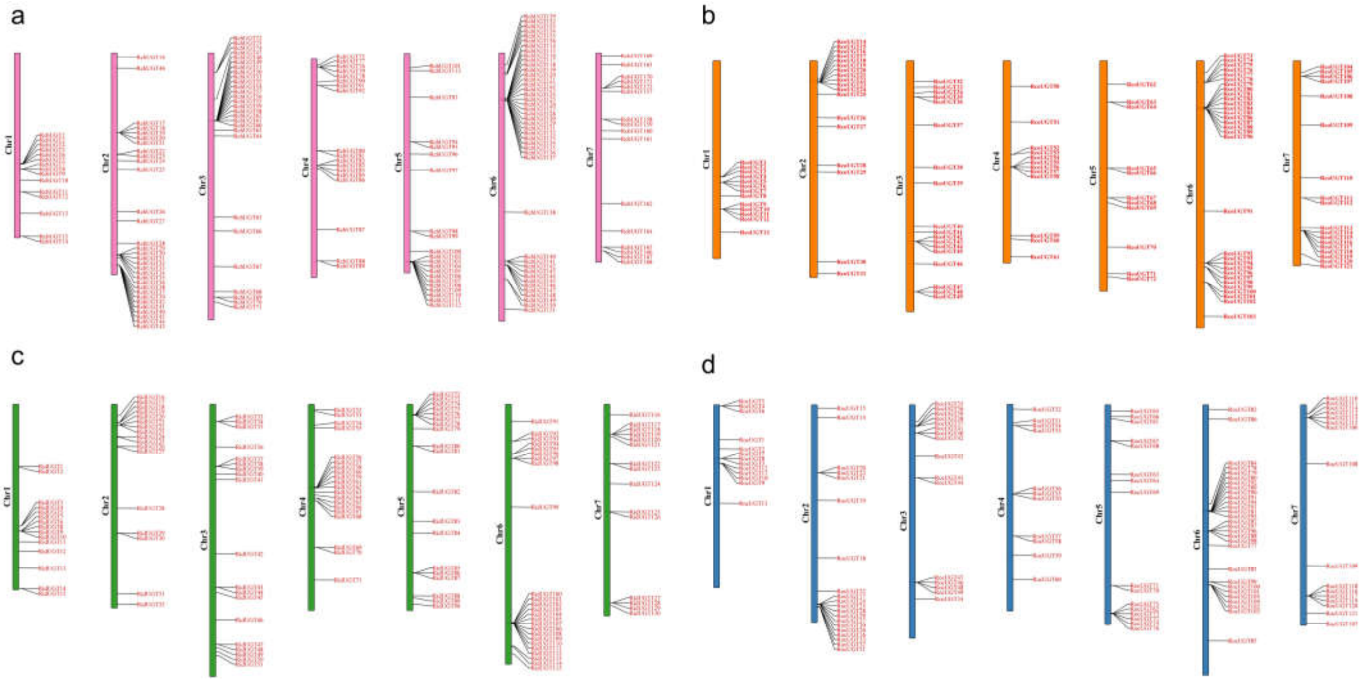

Figure S5 Chromosome Mapping of *UGTs* Gene in four *Rubus* species.

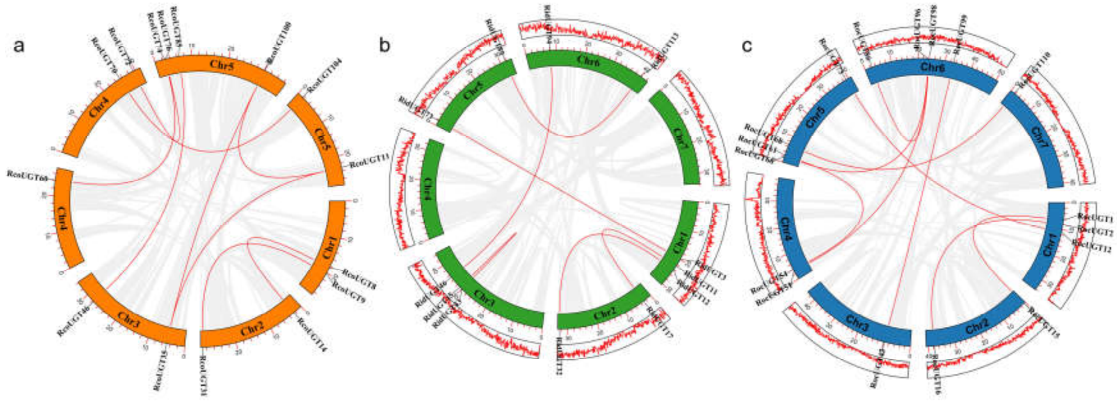

Figure S6 Collinear analysis of *UGTs* gene family from *R. corchorifolius* (a)*, R. idaeus* (b) *and R. occidentalis* (c).

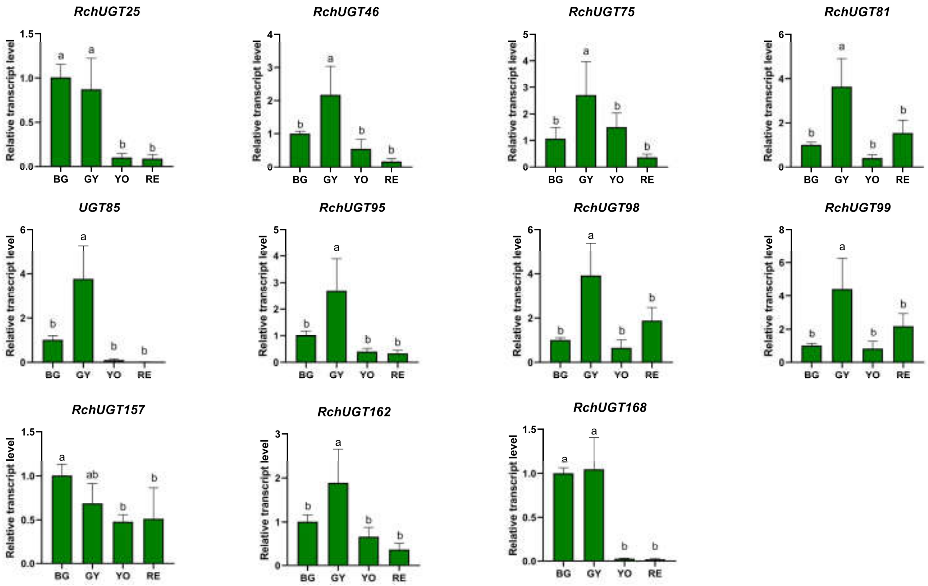

Figure S7 RT-qPCR results of *RchUGTs* in four stages of fruits development. BG (big green, 21 DPA), GY (green-to-yellow, 42 DPA), YO (yellow-to-orange, 48 DAP) and Re (red, 54 DPA).

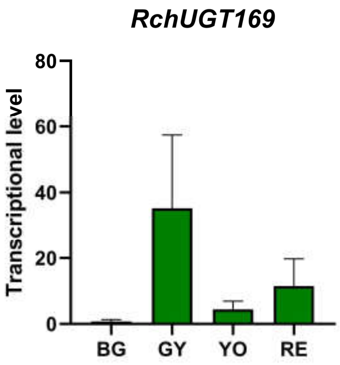

Figure S8 The expression patterns of *RchUGT169* in four stages of fruits development.

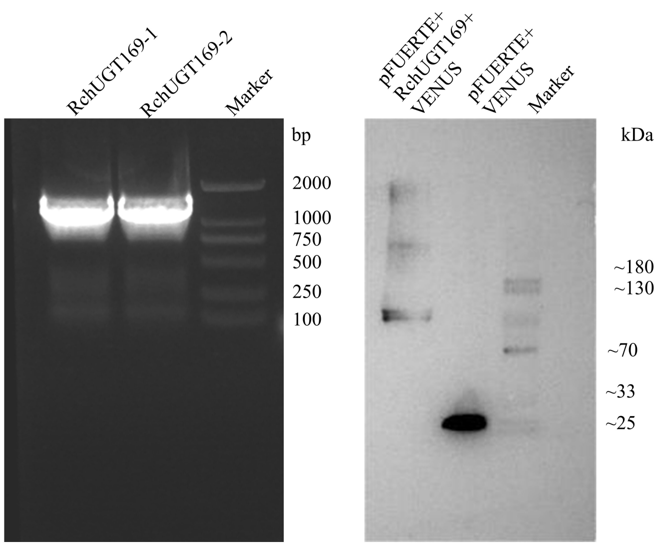

Figure S9 Full-length CDS cloning of *RchUGT169* gene of raspberry and SDS-PAGE of RchUGT169 recombinant protein.
